# Navigating the complexity of WHO CNS5: the evolutionary trajectory of glioma classification and the emergence of large language models

**DOI:** 10.3389/fonc.2026.1867911

**Published:** 2026-07-22

**Authors:** Minghao Lian, Erman Wu, Guohu Kuai, Guohua Zhu, Mijiti Maimaitili, Dangmurenjiafu Geng

**Affiliations:** Neurosurgery Center of the First Affiliated Hospital of Xinjiang Medical University, Urumqi, Xinjiang Uyghur Autonomous Region, China

**Keywords:** artificial intelligence, glioma, integrated diagnosis, large language models, molecular biology, WHO classification

## Abstract

The 2021 World Health Organization (WHO) Classification of Tumors of the Central Nervous System (fifth edition, WHO CNS5) marks a profound paradigm shift from traditional morphologic assessment to a biologically and molecularly integrated diagnostic framework. While this evolution has significantly enhanced diagnostic precision, it has also imposed a substantial cognitive burden on clinicians. This challenge is particularly evident when navigating the fragmented landscape of molecular biomarkers and the increasingly intricate grading logic required for precise classification. This review delineates the historical trajectory of glioma classification standards and evaluates the recent research progress of Large Language Models (LLMs) in assisting neuro-oncological text processing. Evidence suggests that through advanced technologies such as Retrieval-Augmented Generation (RAG) and Reinforcement Learning from Human Feedback (RLHF), LLMs can effectively synthesize unstructured clinical data, mitigate the risk of “hallucinations,” and generate integrated diagnostic recommendations compliant with the latest standards. The transition toward an intelligent diagnostic paradigm is poised to provide critical support for the precise classification and personalized therapeutic closure of gliomas.

## 1 Introduction

Gliomas represent the most prevalent and lethal primary malignancies of the central nervous system (CNS), characterized by high heterogeneity and varying clinical trajectories (1–3). Compared to other malignancies, gliomas are characterized by distinct genetic alterations, epigenetic modifications, unique biological features, and a specialized immune microenvironment. Collectively, these factors contribute to a profound resistance against both conventional and novel immunotherapeutic approaches. Consequently, prognostic advancements for glioma patients have remained markedly limited over the past two decades (4, 5), with the median overall survival (OS) persisting at a mere 12–15 months (6). Historically, precise grading and classification have functioned not only as pivotal metrics for prognostic assessment but also as the essential cornerstone for formulating individualized surgical, radiotherapeutic, and chemotherapeutic strategies. However, as neuro-oncology enters the molecular era, the diagnostic paradigm has undergone a fundamental evolution, transitioning from purely phenotypic descriptions toward a framework of rigorous logical deduction predicated on multi-omic evidence.

Since its inaugural publication in 1979, the World Health Organization (WHO) classification of tumors of the central nervous system (CNS) has undergone multiple iterations, with evaluation criteria evolving from purely histological morphology toward the integrated diagnostic paradigm established in the 2021 fifth edition (WHO CNS5) (7–10). Under this new framework, clinicians are required to concurrently synthesize histological features, immunohistochemical phenotypes, and genomic sequencing data—such as *IDH* mutations, *1p/19q* codeletion, and *CDKN2A/B* homozygous deletion (11). Although this paradigm shift has significantly improved the biological consistency of diagnoses, it has also imposed an unprecedented cognitive burden. The logic of integrated diagnosis entails complex conditional branching and priority adjudication, with critical evidence often existing in an unstructured format scattered across heterogeneous texts, including pathological reports, molecular assays, and surgical records. Traditional clinical pathways, which rely on the manual integration of this fragmented information by physicians, are not only limited in efficiency but also present a significant risk of misinterpretation when navigating intricate diagnostic logics.

**Figure 1:**
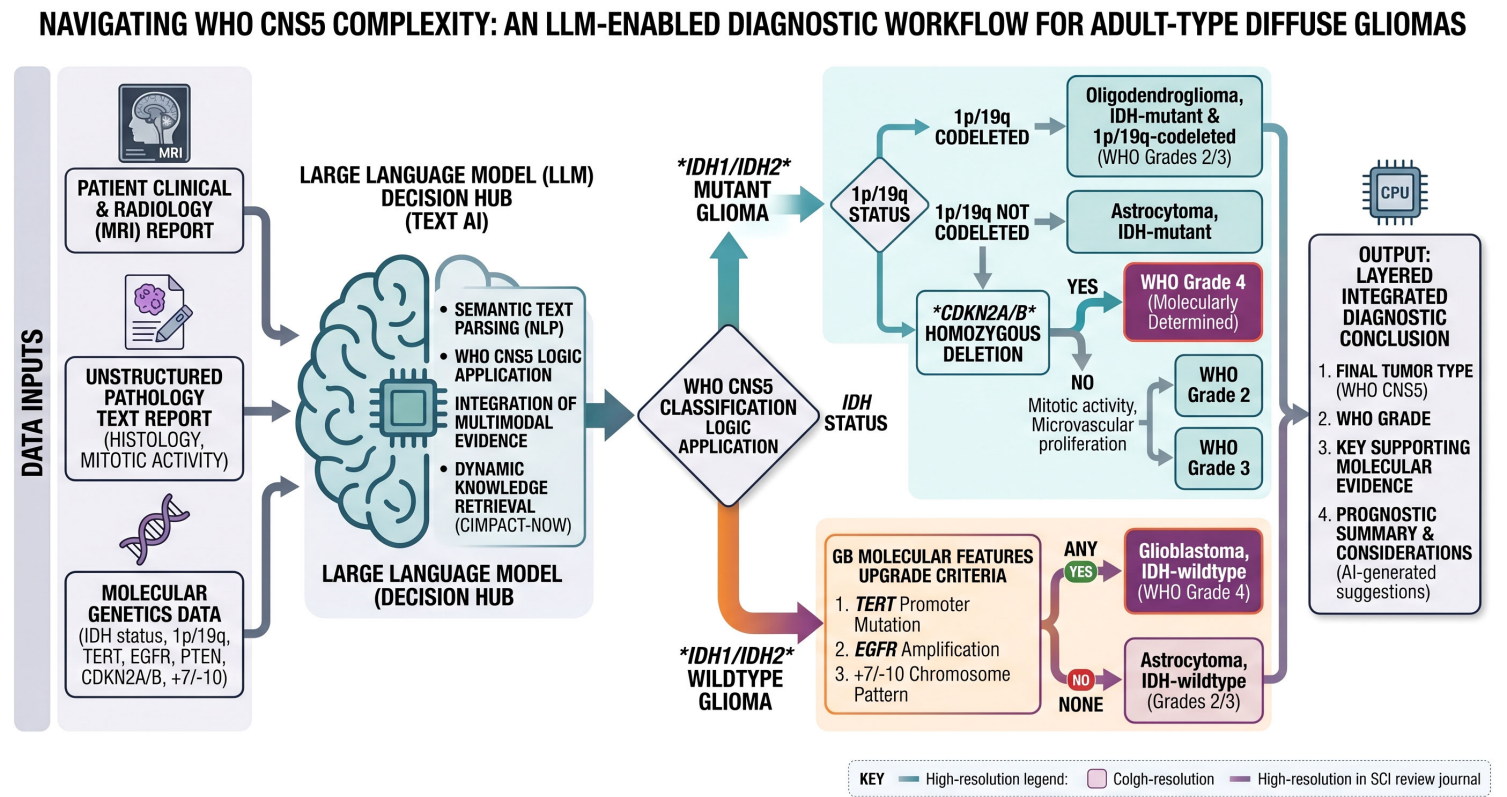
Schematic illustration of the LLM-driven diagnostic and therapeutic framework navigating the complexity of WHO CNS5. **Figure 1** This schematic outlines the logic of Large Language Models (LLMs) in parsing unstructured clinical data and mapping molecular markers (e.g., *IDH*, *1p/19q*, *CDKN2A/B*) to the integrated diagnostic criteria of WHO CNS5. The workflow highlights the transition from fragmented textual evidence to standardized clinical decision-making.

Large Language Models (LLMs), with their emergent capabilities in natural language processing (NLP), exhibit profound potential to bridge the aforementioned cognitive gaps (12). Unlike computer vision-based artificial intelligence that primarily focuses on image feature extraction, LLMs possess the capacity to handle long-range semantic dependencies and perform sophisticated logical deduction. This enables them to replicate the “Chain of Thought” (CoT) of clinical experts, facilitating the automated mapping from unstructured medical text to standardized integrated grading (13–16) (**Figure 1**) AI-driven personalized medicine is expected to significantly enhance the efficacy of oncological therapies and ultimately improve patient outcomes (17, 18). The objective of this review is to systematically delineate the evolutionary trajectory of glioma classification and grading standards, analyze the core mechanisms of LLMs in textual logic parsing, and explore the future directions for the intelligent transformation of neuro-oncological diagnostic and therapeutic models.

This narrative literature review systematically delineates the evolutionary trajectory of glioma classification and grading standards, analyzes the algorithmic mechanisms of large language models (LLMs) in structural logic parsing, and explores future paradigms for the intelligent transformation of neuro-oncological diagnostic and therapeutic workflows. The primary contribution and added value of this manuscript lie in its novel evaluation of LLMs as specialized cognitive enhancers capable of advanced medical reasoning, rather than mere text-processing utilities. Distinct from traditional radiomics-focused reviews, this study establishes a direct conceptual and structural mapping between the hierarchical conditional logic of WHO CNS5 and the emerging Chain of Thought (CoT) reasoning of LLMs, thereby providing a practical blueprint to mitigate clinical cognitive overload.

## 2 Evolution of glioma grading and classification standards (1979–2021)

The foundations of glioma classification were laid in the early 20th century, during which its core logic underwent a profound transformation from gross pathological observation to microscopic histological diagnosis (**Table 1**). In 1926, Bailey and Cushing first proposed a systematic classification of neuroepithelial tumors based on the embryonic residue theory and introduced the concept of “tumor grading” to correlate pathological features with patient prognosis (23). Although this theory had historical limitations in the understanding of cellular differentiation, it established the structural framework for modern glioma classification. Since the publication of the inaugural World Health Organization (WHO) Classification of Tumors of the Central Nervous System in 1979, glioma diagnosis has long adhered to the principle of “histogenesis.” This approach categorizes tumors based on the morphological similarities between neoplastic cells and normal glial cells, as observed through microscopic hematoxylin and eosin (H&E) staining, immunohistochemistry (IHC), and ultrastructural examination. This histology-based system remained the predominant diagnostic paradigm throughout the first four editions of the “Blue Books” from 1979 to 2007 (9, 10, 19–22). During this period, the classification entries evolved from 12 major categories in the first edition to a more refined system; the third edition introduced ICD-O (International Classification of Diseases for Oncology) coding and transitioned toward a closed classification model. Although molecular genetic information began to be mentioned in the third and fourth editions, it served only as supplementary reference indicators for clinical prognosis and had not yet challenged the central authority of histological classification.

**Table 1:** Evolutionary trajectory of glioma diagnostic criteria in the WHO classification of tumors of the central nervous system.

WHO version (year)	Core diagnostic paradigm	Complexity of diagnostic logic	Clinical cognitive challenges	Potential demand for AI/LLM	References
First, Second, and Third Editions (1979, 1993, 2000)	Pure Histological Morphology: Assessment of cellular atypia, mitotic activity, and necrosis under light microscopy.	Low: Linear logic.	Subjective variability among observers.	Extremely Low: Only requires digital imaging support.	(19–21)
Fourth Edition (2007)	Refined Histology: Introduction of auxiliary subtypes and initial focus on genetic background.	Medium: Descriptive logic.	Numerous and overlapping subtypes; difficult to achieve precise prognostic prediction.	Low: Requires structured databases.	(22)
Revised Fourth Edition (2016)	Commencement of the Molecular Era: Mandatory introduction of IDH mutation and 1p/19q status.	High: Branching logic.	Must integrate experimental data; traditional visual assessment becomes insufficient.	Medium: Requires preliminary data processing.	(10)
Fifth Edition (WHO CNS5, 2021)	Integrated Diagnostic Paradigm: Molecular status takes precedence; introduction of “molecular upgrade” concepts.	Extremely High: Complex decision trees.	Cognitive overload: adjudication pathways prone to error when molecular markers conflict with morphology.	Extremely High: Requires LLMs for evidence alignment and logical reasoning.	(9)

With the breakthroughs in molecular biology, the limitations of traditional histological classification—such as poor inter-observer consistency and the inability to accurately predict clinical behavior and therapeutic response—became increasingly evident. These challenges necessitated a paradigm shift toward a molecularly integrated diagnostic framework. The 2016 revised fourth edition of the WHO classification served as a landmark milestone, for the first time mandating the inclusion of molecular features alongside histological assessment. This update shattered the constraints of relying solely on microscopic morphology and implemented integrated diagnosis for entities including diffuse gliomas and medulloblastomas (10). By merging diffuse astrocytic and oligodendroglial tumors and redefining entities based on IDH mutation and 1p/19qcodeletion status, this edition successfully addressed the long-standing ambiguity surrounding mixed gliomas, such as oligoastrocytomas. However, as the rapid pace of molecular oncology outpaced the formal update cycles of the WHO “Blue Books,” the non-official organization cIMPACT-NOW (Consortium to Inform Molecular and Practical Approaches to CNS Tumor Taxonomy - Not Officially WHO) issued eight update recommendations. These guidelines provided critical theoretical support and practical supplements for the subsequent comprehensive overhaul of the classification framework (7, 24).

The 2021 World Health Organization Classification of Tumors of the Central Nervous System (fifth edition, WHO CNS5), reviewed in Neuro-Oncology in late June 2021, represents a more thorough biological transformation. The most transformative change is the formal division of diffuse gliomas into “adult-type” and “pediatric-type” based on their molecular genetic characteristics (9). This classification transcends the conventional scope of age at onset, profoundly reflecting fundamental differences in driver mutations and biological behaviors. For instance, adult-type diffuse gliomas have been streamlined into three definitive entities: Astrocytoma (*IDH*-mutant), Oligodendroglioma (*IDH*-mutant and *1p/19q*-codeleted), and Glioblastoma (*IDH*-wildtype) (9). In the new simplified taxonomy, histological subtypes are no longer presented independently. Instead, the emphasis has shifted toward layered diagnosis—integrating histopathological features with molecular information—while introducing the suffixes NOS (Not Otherwise Specified) and NEC (Not Elsewhere Classified) to standardize reporting when molecular data is unavailable or discordant. This shift not only renders diagnosis more specific and objective but also provides a more homogeneous patient population for individualized therapy based on molecular targets and precise clinical trial enrollment.

Parallel to the evolution of the classification framework, tumor grading standards have undergone significant adjustments, transitioning from purely histological scoring to integrated molecular assessment. The updated WHO classification utilizes Arabic numerals (CNS WHO grades 1–4) instead of Roman numerals (grades I–IV) to minimize transcriptional errors and ensure alignment with the grading standards of other organ systems. Crucially, the new edition introduces the principle of “grading within types” and incorporates molecular grading indicators alongside histological criteria. This implies that even if microvascular proliferation or necrosis is not histologically observed, an Astrocytoma (*IDH*-mutant) or a diffuse Astrocytoma (*IDH*-wildtype) can be directly designated as CNS WHO grade 4 if any of the following molecular alterations are identified: *CDKN2A/B* homozygous deletion, *TERT* promoter mutation, *EGFR* amplification, or a chromosome 7 gain/chromosome 10 loss (+7/-10) pattern (9, 25, 26).The scientific rigor of this grading system stems from the deep integration of natural history and treatment response, aiming to provide objective, prognostic guidance for individualized oncology therapeutics.

## 3 Cognitive burden of integrated molecular diagnosis and the opportunities for LLM intervention

With the deep implementation of WHO CNS5, glioma diagnosis has transitioned from a solitary morphological evaluation to an exceedingly complex “matrix matching” paradigm. This integrated diagnostic system, which synthesizes histopathological phenotypes with multi-dimensional molecular features (e.g., *IDH* mutation, *1p/19q* codeletion, and *CDKN2A/B* homozygous deletion), has imposed an unprecedented cognitive burden on clinicians (9). Against a backdrop where the boundaries between adult-type and pediatric-type gliomas are increasingly distinct and molecular subgroups are becoming further subdivided, physicians must not only accurately interpret complex molecular testing reports but also track dynamic updates from non-official organizations such as cIMPACT-NOW in real-time. This is essential to avoid diagnostic biases, particularly when navigating “Not Otherwise Specified” (NOS) or “Not Elsewhere Classified” (NEC) designations (27–29). The highly fragmented and rapidly evolving nature of this knowledge system makes manual information integration and classification decision-making increasingly difficult. Consequently, there is an urgent need for advanced natural language processing (NLP) capabilities to assist clinicians in achieving precise mapping from raw laboratory data to standardized integrated diagnoses (**Table 2**).

**Table 2:** Comparative summary of LLM-based and AI-driven models relevant to oncology text processing and classification.

Model/study	Data inputs	Model architecture	Performance metrics	Dataset size	Key limitations
Med-PaLM 2/GPT-4 Iterations (36)	Unstructured histopathology & clinical reports	Large-scale generative Transformer	High sensitivity in extracting biomarkers (IDH, 1p/19q)	Hundreds of multi-center reports	Vulnerable to hallucinating rare subtypes; lack of real-time clinical reasoning verification.
BioMedLM/ChatDoctor (35)	MR radiology text reports & medical consults	Domain-specific fine-tuned LLM	Accuracy approaching human experts in text cleaning	Large-scale medical corpus	Strictly text-based; struggles with spatial alignment of multi-modal features.
AdaBoost Framework (38)	Structured blood tests & laboratory metrics	Ensemble machine learning	Metastasis prediction AUROC: 0.942 (vs. Clinicians 0.815)	Patient cohort data	Limited semantic processing capability; cannot parse unstructured narrative text.
NER Staging Framework (39)	Semi-structured clinical database records (TCGA-THCA)	Named Entity Recognition + LLM	Optimized staging and risk classification efficiency	High-throughput public dataset	High dependence on data standardization; poor generalizability to messy, real-world notes.

In the technological landscape of AI-empowered neuro-oncology, a fundamental distinction exists between LLM-based “textual AI” and traditional “imaging AI” regarding their architectural design and application domains. The former focuses on medical logical reasoning and the semantic synthesis of unstructured clinical data (30). While imaging AI primarily extracts pixel-level features to assist in diagnosis and tumor segmentation, textual AI specializes in processing linguistic symbols, such as pathological descriptions, molecular sequencing data, and vast bodies of medical literature. Its core value lies in understanding and applying complex classification standards (31–33). Since the final diagnosis of glioma often relies on the logical combination of textual narratives and molecular indicators in pathology reports—rather than pure image recognition—LLMs demonstrate unique domain advantages in parsing complex classification guidelines, integrating multi-source textual evidence, and generating standardized, layered diagnostic recommendations.

Current research on LLMs in the field of glioma is transitioning from general-purpose models to domain-specific fine-tuning. The research focus is centered on the models’ “zero-shot” or “few-shot” reasoning capabilities regarding complex pathological criteria. By pre-training models on large-scale medical corpora, researchers enable them to recognize subtle nomenclature changes and grading parameter weights in glioma classification. This allows the models to provide logically consistent diagnostic inferences based on levels of evidence, even when dealing with incomplete or contradictory clinical texts (11, 34). By simulating the logical reasoning of neuropathologists, this content-based AI technology can effectively identify core biological markers within pathology reports and automatically associate them with CNS WHO grades. This not only enhances diagnostic consistency but also significantly accelerates the clinical decision-making cycle.

## 4 Current research status of large language models in glioma text processing and auxiliary classification

In the ongoing advancement of intelligent neuro-oncological diagnosis and treatment, LLMs are progressively evolving from general-purpose corpora processing to the precise interpretation of pathological texts. Their core advantage lies in bridging the cognitive gap between complex molecular terminology and clinical classification standards. Current medical-domain models, such as Med-PaLM, BioMedLM, and ChatDoctor, have demonstrated diagnostic accuracy in medical consultation and medical record analysis that approaches or even exceeds that of human physicians through large-scale data training (35) Recent studies have indicated that LLMs fine-tuned for vertical medical domains, such as Med-PaLM2 or specific iterations of GPT-4, exhibit remarkable sensitivity in interpreting unstructured pathology reports and extracting pivotal biomarkers like IDH mutations and 1p/19q codeletion (36). Prospective cases suggest that through Retrieval-Augmented Generation (RAG) technology, LLMs can retrieve real-time cIMPACT-NOW update recommendations and logically cross-reference them with patient pathological descriptions. This effectively mitigates the mnemonic burden and decision bias of clinicians facing the continuously updating WHO CNS5 standards (24, 37). To provide a broader technological context for oncological text processing, benchmarking non-glioma AI applications offers valuable cross-disciplinary insights. For instance, in a study based on blood test results, the AdaBoost model performed excellently in predicting metastasis in patients with pheochromocytoma and paraganglioma, with an Area Under the Receiver Operating Characteristic curve (AUROC) reaching 0.942, surpassing the 0.815 achieved by clinical experts (38). Furthermore, demonstrating the generalizability of language architectures in oncology, a named entity recognition (NER) framework has been developed to extract semi-structured clinical record information from The Cancer Genome Atlas-Thyroid Cancer (TCGA-THCA) database, optimizing the efficiency and accuracy of staging and risk classification for thyroid cancer patients (39). These external algorithmic paradigms provide foundational structural blueprints that can be directly translated to optimize the semantic processing of unstructured glioma pathology matrices. Such semantic-based auxiliary systems, by simulating the expert CoT reasoning, can not only accurately identify complex elements of integrated diagnosis but also automatically prompt clinicians to focus on molecular details with significant prognostic value (40, 41).

Current research on text-based AI for glioma has advanced beyond simple keyword extraction toward the comprehension and generation of complex medical logic, particularly in handling highly heterogeneous clinical texts. For instance, pioneering studies have utilized LLMs to perform automated cleaning and standardized mapping of multi-center glioma pathology reports. These models can precisely distinguish subtle clinical descriptive nuances between IDH-wildtype and mutant states through contextual analysis, converting dispersed immunohistochemistry results into integrated diagnostic conclusions compliant with WHO specifications (41–43). This textual AI complements imaging AI—which focuses on spatial feature extraction—by emphasizing the logical translation of medical consensus, thereby providing an efficient technological pathway for resolving the fragmentation of textual information common in clinical diagnosis.

In current vertical domain explorations, LLMs have demonstrated unique potential in predicting glioma disease trajectories and designing auxiliary clinical pathways. By integrating historical radiological descriptions, pathological texts, and longitudinal follow-up records, these models utilize natural language generation (NLG) technology to provide detailed prognostic analysis and issue early grading alerts for high-risk molecular features, such as *CDKN2A*/B homozygous deletion (32, 44). Existing research has leveraged LLMs to parse the textual dimensions of massive multimodal datasets, facilitating the discovery of correlations between specific linguistic patterns—which are often unidentifiable by traditional statistics—and the risk of glioma recurrence (32, 37). Notably, Innani S. et al. recently developed an AI-driven model capable of diagnosing gliomas according to the WHO 2021 classification based solely on H&E-stained whole-slide images (WSI) (45). Such research based on large-scale clinical language models not only deepens medical data mining but also significantly narrows the time window from molecular data acquisition to definitive clinical intervention by automating the generation of standardized diagnostic reports.

Despite the rapid research advancements of LLMs in the field of glioma, more rigorous verification strategies are required to ensure diagnostic interpretability and mitigate the risk of medical knowledge “hallucinations.” Current academic frontiers are dedicated to constructing glioma-specific prompt frameworks that guide models to provide diagnostic classification suggestions while explicitly citing specific clauses of the WHO CNS5 guidelines. Through the incorporation of Reinforcement Learning from Human Feedback (RLHF) combined with real-time calibration by professional physicians, the robustness of these models in handling complex and rare subtypes has been significantly enhanced. Such evidence-chain-oriented AI auxiliary systems not only provide a paradigm for the future popularization of intelligent neuro-oncological classification standards but also establish a textual foundation for cross-institutional clinical collaboration and the construction of high-quality epidemiological databases (37, 46, 47).

However, a critical appraisal of the current literature indicates that existing evidence remains predominantly descriptive and is constrained by several fundamental bottlenecks. First, divergent outcomes across varying model scales highlight pronounced heterogeneity in performance metrics; specifically, localized fine-tuning on smaller architectures frequently compromises generalizability in favor of narrow accuracy. Second, systemic dataset biases persist, as most contemporary models are trained on highly curated, public repositories (e.g., TCGA) or structured multi-center registries, thereby failing to replicate the unstructured, fragmented, and incomplete nature of real-world clinical documentation. Third, the intrinsic ‘black-box’ architecture of LLMs poses formidable interpretability challenges, impeding the ability of clinicians to delineate the exact causal reasoning chain underlying a specific molecularly driven upgrade recommendation. Lastly, clinical translation is hindered by stringent deployment barriers, including rigorous validation protocols, data privacy mandates, and the pronounced vulnerability of LLMs to adversarial hallucination attacks within clinical decision-support systems (47). Consequently, transitioning from speculative, proof-of-concept cases to robust, bedside-ready tools necessitates the establishment of standardized evaluation benchmarks and rigorous physician-in-the-loop calibration frameworks.

## 5 Perspectives and outlook

While text-based LLMs and radiomics-driven imaging AI currently operate on relatively independent technological trajectories, the inevitable trend in precision neuro-oncology is moving toward the deep semantic fusion of cross-modal data. The WHO CNS5 has established a framework for integrated diagnosis, necessitating that clinicians evaluate molecular features alongside histopathological assessment. Future intelligent decision-making hubs will transcend the mere processing of unstructured pathological descriptions; instead, they will achieve a logical alignment across multimodal perceptions—integrating diffuse infiltrative patterns from imaging, cellular differentiation levels from histology, and driver gene alterations from multi-omic sequences. This “ultra-integrated” diagnostic paradigm will leverage the high-dimensional computational advantages of AI to identify biological consistencies imperceptible to the human eye or traditional statistics. By constructing a precise “digital pathological portrait” for every patient, this approach not only aligns with the new standards set by the WHO for clinical and basic science research but also serves as the core engine driving the paradigm shift in neuro-oncology.

Given that progress in molecular oncology far outpaces the update cycles of official guidelines, LLMs will function as a digital bridge connecting the dynamic frontiers of scientific research with real-time clinical decision-making. The WHO classification system is viewed as an evolving process, a characteristic that demands high clinical flexibility to absorb the latest evidence. Future AI systems will be capable of parsing update recommendations released by cIMPACT-NOW in real-time, translating them into directly applicable auxiliary diagnostic logic. Such intelligent platforms with continuous learning capabilities can effectively eliminate the cognitive lag of clinicians regarding complex new entities, ensuring that even primary medical institutions can adhere to the world’s most advanced diagnostic and therapeutic guidelines. This will significantly enhance the level of homogenization and objectivity during the execution of glioma grading and classification standards.

The application of intelligent technologies will drive the transition of glioma management from simple classification diagnosis to a precision medicine “closed-loop,” demonstrating immense potential particularly in the screening of molecularly targeted drugs and precise enrollment in clinical trials. The new classification underscores the importance of defining tumor entities based on biological and molecular characteristics, with the ultimate goal of improving patient survival and fostering individualized medicine. Future LLMs will be able to automatically match optimal treatment strategies or ongoing clinical trials based on a patient’s specific molecular alterations. Particularly when addressing pediatric-type diffuse gliomas—which exhibit low incidence and complex molecular backgrounds—intelligent systems can assist physicians in finding a balance between tumor heterogeneity and therapeutic targets, optimizing the efficacy of clinical trials, and utilizing molecular biomarkers to dynamically monitor treatment response. This will truly achieve individualized precision intervention based on the natural history and biological essence of the disease.

In the future paradigm of human-AI collaboration, LLMs will evolve into logic verifiers and workflow optimizers within neuro-oncology, aiming to minimize the subjective bias introduced by complex grading systems. Although histological diagnosis remains a pivotal “gold standard,” its inherent ambiguity and inter-observer variability have prompted the inclusion of molecular parameters in grading standards. By parsing key terminology in pathological reports, AI systems can automatically associate molecular indicators according to CNS WHO grading principles, ensuring that diagnostic results strictly comply with the latest academic specifications. This intelligent transformation is not intended to replace professional physicians; rather, by sharing the burden of high-intensity cognitive load, it enables neuropathologists and neurosurgeons to shift their focus toward more creative analysis of challenging cases and humanistic patient care. This future vision of enhancing diagnostic precision and prognostic predictive power through technological empowerment will provide a solid technical foundation for the leapfrog development of neuro-oncological care worldwide.

Furthermore, actualizing this intelligent paradigm requires rigorous interdisciplinary collaboration and cross-domain knowledge integration between clinicians and computer scientists. Neuropathologists and neurosurgeons must acquire fundamental AI literacy—specifically regarding prompt engineering and model interpretability—while computer scientists must comprehend the clinical complexities of neuro-oncology. Enhancing this mutual literacy ensures that model development addresses practical clinical requirements while maintaining diagnostic accuracy. Consequently, joint institutional training and cross-disciplinary task forces are essential to formalize this integration, effectively transforming LLMs into functional components of the clinical workflow.

## References

[1] Horbinski C Solomon DA Lukas RV Packer RJ Brastianos P Wen PY . Molecular testing for the World Health Organization classification of central nervous system tumors: a review. JAMA Oncol. (2025) 11:317–28. doi: 10.1001/jamaoncol.2024.5506 39724142 PMC12344474

[2] Van Den Bent MJ Geurts M French PJ Smits M Capper D Bromberg JEC . Primary brain tumours in adults. Lancet. (2023) 402:1564–79. doi: 10.1016/s0140-6736(23)01054-1 37738997

[3] Price M Neff C Nagarajan N Kruchko C Waite KA Cioffi G . CBTRUS statistical report: American Brain Tumor Association & NCI Neuro-Oncology Branch adolescent and young adult primary brain and other central nervous system tumors diagnosed in the United States in 2016-2020. Neuro Oncol. (2024) 26:iii1–iii53. doi: 10.1093/neuonc/noae047 38709657 PMC11073545

[4] Horbinski C Berger T Packer RJ Wen PY . Clinical implications of the 2021 edition of the WHO classification of central nervous system tumours. Nat Rev Neurol. (2022) 18:515–29. doi: 10.1038/s41582-022-00679-w 35729337

[5] Chai RC Liu X Pang B Liu YQ Li JJ Li YF . Recurrent PTPRZ1-MET fusion and a high occurrence rate of MET exon 14 skipping in brain metastases. Cancer Sci. (2022) 113:796–801. doi: 10.1111/cas.15211 34812554 PMC8819346

[6] Clavreul A Lemée JM Soulard G Rousseau A Menei P . A simple preoperative blood count to stratify prognosis in isocitrate dehydrogenase-wildtype glioblastoma patients treated with radiotherapy plus concomitant and adjuvant temozolomide. Cancers (Basel). (2021) 13:4416. doi: 10.3390/cancers13225778 34830935 PMC8616081

[7] Yang XJ Jiang T Chen ZP Yu SZ . Evolution of the World Health Organization classification of tumors of the central nervous system: 1979-2021. Chin J Contemp Neurol Neurosurg. (2021) 21:710–24.

[8] Sejda A Grajkowska W Trubicka J Szutowicz E Wojdacz T Kloc W . WHO CNS5 2021 classification of gliomas: a practical review and road signs for diagnosing pathologists and proper patho-clinical and neuro-oncological cooperation. Folia Neuropathologica. (2022) 60:137–52. doi: 10.5114/fn.2022.118183 35950467

[9] Louis DN Perry A Wesseling P Brat DJ Cree IA Figarella-Branger D . The 2021 WHO classification of tumors of the central nervous system: a summary. Neuro Oncol. (2021) 23:1231–51. doi: 10.1093/neuonc/noab106 34185076 PMC8328013

[10] Louis DN Perry A Reifenberger G von Deimling A Figarella-Branger D Cavenee WK . The 2016 World Health Organization classification of tumors of the central nervous system: a summary. Acta Neuropathol. (2016) 131:803–20. doi: 10.1007/s00401-016-1545-1 27157931

[11] Bruford EA Braschi B Denny P Jones TEM Seal RL Tweedie S . Guidelines for human gene nomenclature. Nat Genet. (2020) 52:754–8. doi: 10.1038/s41588-020-0669-3 32747822 PMC7494048

[12] Omar M Nadkarni GN Klang E Glicksberg BS . Large language models in medicine: a review of current clinical trials across healthcare applications. PloS Digit Health. (2024) 3:e0000662. doi: 10.1371/journal.pdig.0000662 39561120 PMC11575759

[13] Bitterman DS Miller TA Mak RH Savova GK . Clinical natural language processing for radiation oncology: a review and practical primer. Int J Radiat Oncol Biol Phys. (2021) 110:641–55. doi: 10.1016/j.ijrobp.2021.01.044 33545300

[14] Loor-Torres R Wu Y Esteban Cabezas null Borras-Osorio M Toro-Tobon D Duran M . Use of natural language processing to extract and classify papillary thyroid cancer features from surgical pathology reports. Endocr Pract. (2024) 30:1051–8. doi: 10.1016/j.eprac.2024.08.008 39197747 PMC11531997

[15] Rajaganapathy S Chowdhury S Li X Buchner V He Z Zhang R . Synoptic reporting by summarizing cancer pathology reports using large language models. npj Health Syst. (2025) 2:11. doi: 10.1038/s44401-025-00013-8 41623418 PMC12858025

[16] Maimone C Dolan BM Green MM Sanguino SM O'Brien CL . Using natural language processing to visualize narrative feedback in a medical student performance dashboard. Acad Med. (2024) 99:1094–8. doi: 10.1097/acm.0000000000005800 38967963

[17] Rezayi S S R N K Saedi S . Effectiveness of artificial intelligence for personalized medicine in neoplasms: a systematic review. BioMed Res Int. (2022) 2022:7842566. doi: 10.1155/2022/7842566 35434134 PMC9010213

[18] Shao Y Lv X Ying S Guo Q . Artificial intelligence-driven precision medicine: multi-omics and spatial multi-omics approaches in diffuse large B-cell lymphoma (DLBCL). Front Biosci (Landmark Ed). (2024) 29:404. doi: 10.31083/j.fbl2912404 39735973

[19] Zülch KJ . Principles of the new World Health Organization (WHO) classification of brain tumors. Neuroradiology. (1980) 19:59–66. doi: 10.1007/bf00342596 6245388

[20] Kleihues P Burger PC Scheithauer BW . The new WHO classification of brain tumours. Brain Pathol. (1993) 3:255–68. doi: 10.1111/j.1750-3639.1993.tb00752.x 8293185

[21] Kleihues P Louis DN Scheithauer BW Rorke LB Reifenberger G Burger PC . The WHO classification of tumors of the nervous system. J Neuropathol Exp Neurol. (2002) 61:215–25. doi: 10.1093/jnen/61.3.215 11895036

[22] Louis DN Ohgaki H Wiestler OD Cavenee WK Burger PC Jouvet A . The 2007 WHO classification of tumours of the central nervous system. Acta Neuropathol. (2007) 114:97–109. doi: 10.1007/s00401-007-0243-4 17618441 PMC1929165

[23] Yang XJ . Interpretation of the evolution of histological classification of central nervous system tumors from a neurosurgeon's perspective. Chin J Contemp Neurol Neurosurg. (2008) 8:376–83.

[24] Sahm F Aldape KD Brastianos PK Brat DJ Dahiya S von Deimling A . cIMPACT-NOW update 8: clarifications on molecular risk parameters and recommendations for WHO grading of meningiomas. Neuro Oncol. (2025) 27:319–30. doi: 10.1093/neuonc/noae170 39212325 PMC11812049

[25] Yang XJ Yin HF Li Z Yu SZ . Chinese version of simplified table of 2021 WHO classification of tumors of the central nervous system (fifth edition) and translational interpretations. Chin J Contemp Neurol Neurosurg. (2021) 21:746–50.

[26] Wang L Pan YW Qu Y Gong L . Interpretation on adult type diffuse gliomas in the 2021 WHO classification of tumors of the central nervous system (fifth edition). Chin J Contemp Neurol Neurosurg. (2021) 21:783–90.

[27] Louis DN Wesseling P Paulus W Giannini C Batchelor TT Cairncross JG . cIMPACT-NOW update 1: not otherwise specified (NOS) and not elsewhere classified (NEC). Acta Neuropathol. (2018) 135:481–4. doi: 10.1007/s00401-018-1808-0 29372318

[28] Louis DN Wesseling P Aldape K Brat DJ Capper D Cree IA . cIMPACT-NOW update 6: new entity and diagnostic principle recommendations of the cIMPACT-Utrecht meeting on future CNS tumor classification and grading. Brain Pathol. (2020) 30:844–56. doi: 10.1111/bpa.12832 32307792 PMC8018152

[29] Fujimoto K Arita H Satomi K Yamasaki K Matsushita Y Nakamura T . TERT promoter mutation status is necessary and sufficient to diagnose IDH-wildtype diffuse astrocytic glioma with molecular features of glioblastoma. Acta Neuropathol. (2021) 142:323–38. doi: 10.1007/s00401-021-02337-9 34148105

[30] Luckett PH Olufawo M Lamichhane B Park KY Dierker D Verastegui GT . Predicting survival in glioblastoma with multimodal neuroimaging and machine learning. J Neuro-Oncol. (2023) 164:309–20. doi: 10.1007/s11060-023-04439-8 37668941 PMC10522528

[31] Qi X Zhang F . Application progress of multimodal MRI combined with artificial intelligence model in the prediction of glioma molecular typing. J Imaging Res Med Appl. (2026) 10:4–6. doi: 10.20267/j.issn.2096-3807.2026.02.002

[32] Long SZ Li PA Liu WJ Yang R Qi XH Zhou Y . Research progress of artificial intelligence in glioma disease trajectory prediction. J Med Inf. (2026) 47:17–24.

[33] Li S Fang X Jin Y Deng Y Hu W Wu B . Improving diagnostic accuracy in preoperative glioma classification: performance of knowledge-enhanced large language models compared with radiologists. NPJ Precis Oncol. (2025) 9:383. doi: 10.1038/s41698-025-01171-6 41310391 PMC12660956

[34] Christodoulou RC Papageorgiou PS Lucio Pereira AC Solomou EE Papageorgiou SG Vassiliou E . Large language models in patient education for brain tumors: opportunities, risks, and ethical considerations. Front Oncol. (2026) 16:1795441. doi: 10.3389/fonc.2026.1795441 41952677 PMC13053261

[35] Kim M Ong KT Choi S Yeo J Kim S Han K . Natural language processing to predict isocitrate dehydrogenase genotype in diffuse glioma using MR radiology reports. Eur Radiol. (2023) 33:8017–25. doi: 10.1007/s00330-023-10061-z 37566271

[36] Truhn D Loeffler CM Müller-Franzes G Nebelung S Hewitt KJ Brandner S . Extracting structured information from unstructured histopathology reports using generative pre-trained transformer 4 (GPT-4). J Pathol. (2024) 262:310–9. doi: 10.1002/path.6232 38098169

[37] Almaabreh O Al-Dafi R Tabassum A Othman A Abd-Alrazaq A . The performance of artificial intelligence in classifying molecular markers in adult-type gliomas using histopathological images: systematic review. J Med Internet Res. (2026) 28:e78377. doi: 10.2196/78377 41824546 PMC12986776

[38] Wei W Shao J Lyu RQ Hemono R Ma X Giorgio J . Enhanced language models for predicting and understanding HIV care disengagement: a case study in Tanzania. NPJ Digit Med. (2026) 9:165. doi: 10.1038/s41746-026-02349-3 41565873 PMC12909982

[39] Fung MMH Tang EHM Wu T Luk Y Au ICH Liu X . Developing a named entity framework for thyroid cancer staging and risk level classification using large language models. NPJ Digit Med. (2025) 8:134. doi: 10.1038/s41746-025-01528-y 40025285 PMC11873034

[40] Ji Y Yu Z Wang Y . Assertion detection in clinical natural language processing using large language models. In: Proc (Ieee Int Conf Healthc Inform) New York, NY: IEEE, vol. 2024. (2024). p. 242–7. doi: 10.1109/ichi61247.2024.00039 PMC11908446 40092287

[41] Mohammadzadeh I Hajikarimloo B Niroomand B Eini P Ghanbarnia R Habibi MA . Application of artificial intelligence in forecasting survival in high-grade glioma: systematic review and meta-analysis involving 79,638 participants. Neurosurg Rev. (2025) 48:240. doi: 10.1007/s10143-025-03419-y 39954167

[42] Valerio AG Trufanova K de Benedictis S Vessio G Castellano G . From segmentation to explanation: generating textual reports from MRI with LLMs. Comput Methods Programs BioMed. (2025) 270:108922. doi: 10.1016/j.cmpb.2025.108922 40633400

[43] Alsaedi A Alsharif W Gareeballah A Alshoabi S Alhazmi F Alshamrani K . Assessment of the emerging role of AI in diagnosing gliomas using MRI: systematic review and meta-analysis. Neuro-Oncol Adv. (2025) 7:vdaf162. doi: 10.1093/noajnl/vdaf162 40980443 PMC12448723

[44] Fathallah-Shaykh HM Sotoudeh H Bredel M Whitley A Kim J Morón FE . Diagnosing growth in low-grade gliomas with and without artificial intelligence-measured longitudinal volume measurements: a retrospective observational study. Neuro-Oncol Adv. (2026) 8:vdaf271. doi: 10.1093/noajnl/vdaf271 41710546 PMC12909261

[45] Innani S Pitarch-Abaigar C Marwan MM Harmsen H Bell WR Makris D . AI-based prognostic risk stratification of adult-type diffuse gliomas using H&E-stained slides. Neuro-Oncology. (2025) 27:v257–v. doi: 10.1093/neuonc/noaf201.1021

[46] Caglayan A Slusarczyk W Rabbani RD Ghose A Papadopoulos V Boussios S . Large language models in oncology: revolution or cause for concern? Curr Oncol. (2024) 31:1817–30. doi: 10.3390/curroncol31040137 38668040 PMC11049602

[47] Omar M Sorin V Collins JD Reich D Freeman R Gavin N . Multi-model assurance analysis showing large language models are highly vulnerable to adversarial hallucination attacks during clinical decision support. Commun Med (Lond). (2025) 5:330. doi: 10.1038/s43856-025-01021-3 40753316 PMC12318031

